# A spatial-mechanistic model to estimate subnational tuberculosis burden with routinely collected data: An application in Brazilian municipalities

**DOI:** 10.1371/journal.pgph.0000725

**Published:** 2022-09-21

**Authors:** Melanie H. Chitwood, Layana C. Alves, Patrícia Bartholomay, Rodrigo M. Couto, Mauro Sanchez, Marcia C. Castro, Ted Cohen, Nicolas A. Menzies

**Affiliations:** 1 Department of Epidemiology of Microbial Diseases, Yale School of Public Health, Haven, Connecticut, United States of America; 2 Chronic and Airborne Diseases Surveillance Coordination, Ministry of Health, Rio de Janeiro, Brazil; 3 Department of Tropical Medicine, University of Brasília, Brasilia, Brazil; 4 Department of Global Health and Population, Harvard T.H. Chan School of Public Health, Mumbai, India; PLOS: Public Library of Science, UNITED STATES

## Abstract

Reliable subnational estimates of TB incidence would allow national policy makers to focus disease control resources in areas of highest need. We developed an approach for generating small area estimates of TB incidence, and the fraction of individuals missed by routine case detection, based on available notification and mortality data. We demonstrate the feasibility of this approach by creating municipality-level burden estimates for Brazil. We developed a mathematical model describing the relationship between TB incidence and TB case notifications and deaths, allowing for known biases in each of these data sources. We embedded this model in a regression framework with spatial dependencies between local areas, and fitted the model to municipality-level case notifications and death records for Brazil during 2016–2018. We estimated outcomes for 5568 municipalities. Incidence rate ranged from 8.6 to 57.2 per 100,000 persons/year for 90% of municipalities, compared to 44.8 (95% UI: 43.3, 46.8) per 100,000 persons/year nationally. Incidence was concentrated geographically, with 1% of municipalities accounting for 50% of incident TB. The estimated fraction of incident TB cases receiving diagnosis and treatment ranged from 0.73 to 0.95 across municipalities (compared to 0.86 (0.82, 0.89) nationally), and the rate of untreated TB ranged from 0.8 to 72 cases per 100,000 persons/year (compared to 6.3 (4.8, 8.3) per 100,000 persons/year nationally). Granular disease burden estimates can be generated using routine data. These results reveal substantial subnational differences in disease burden and other metrics useful for designing high-impact TB control strategies.

## Introduction

Tuberculosis (TB) is a leading infectious cause of death globally [1] despite the availability of effective combination drug regimens. A major contributor to ongoing TB mortality is the large fraction of cases that are not detected, and therefore not treated. In 2019, the World Health Organization (WHO) estimated that 29% (21–36%) of incident TB cases did not get diagnosed or receive treatment [1]. More effective approaches for case finding, diagnosis and linkage to care are needed to reduce the fraction of TB case that do not receive treatment [2], which contributes to TB morbidity and onward TB transmission.

While there is only limited evidence supporting untargeted active TB case finding activities as an effective strategy to reduce TB transmission in communities [3], trials [4] and modeling studies [5] suggest that active TB case finding interventions targeted to highest risk sub-populations and spatial areas of disease concentration could have a sizeable epidemiologic impact. Identifying spatial areas of TB disease concentration, and especially areas where rates of undiagnosed TB may be high, remains a substantial challenge. In most settings, it is difficult to determine whether spatial heterogeneity in notification rates can be attributed to actual differences in TB incidence or to differences in surveillance system quality. This variability in the quality and completeness of surveillance systems make it difficult to reliably estimate subnational TB disease incidence and the local rates of undiagnosed TB.

Several approaches have been developed to describe subnational patterns of TB disease burden. These include methods that apply spatial smoothing methods to TB case notification data directly (not addressing the problem of under-detection) [6], use expert input and programmatic data to decompose national incidence esimates to local areas [7,8], or implicity assume that case detection rates are constant across modeled locations [9]. In this paper, we describe extensions to a published method for estimating subnational TB burden [10], which allows stable estimates to be generated at a fine geographic scale. Under this approach, local area estimates of TB incidence and the completeness of case detection are generated using a spatial-mechanistic model fit to routine data on TB case notifications and reported deaths.

We demonstrate the feasibility of this approach using data from Brazil. The WHO estimates that Brazil had a national TB incidence of 45 (39–52) per 100,000 in 2018, with a case detection rate of 87% (75% - 100%) [1]. Routine reporting for Brazil demonstrate substantial variation in notification rates across districts, and past studies employing simpler versions of the methods used in this study have suggested considerable subnational variation in incidence and case detection rates [10,11]. In this study, we report estimates of TB incidence, the fraction of cases treated, and the rate of untreated TB disease for each of 5568 contiguous municipalities in the country over the period 2016–2018.

## Methods

### Overview

We developed a novel method for generating small-area TB incidence estimates based on a previous mathematical model of TB incidence and case detection [10]. We made three specific changes to this previous work in order to produce small-area estimates: 1) we incorporated the spatial structure of municipalities to allow smoothing of noisy estimates across neighboring areas; 2) we aggregated notifications over a three-year period to smooth year-to-year stochastic variation at the municipal-level; and 3) we made adjustments to the previous method used to estimate the probability of death during TB treatment.

A small set of sociodemographic predictors were also included in the model to allow for correlations between municipalities with similar characteristics. The model estimates incidence as the sum of individuals with TB who initiate treatment, die without initiating treatment, or ‘self-cure’ without initiating treatment. Accounting for these pathways, we fit the model to the observed number of individuals with TB who receive treatment and the observed number of TB deaths in each of Brazil’s 5568 contiguous municipalities.

### Data

We accessed tuberculosis case notifications from 2016 to 2018 (n = 276,915) from Brazil’s Notifiable Diseases Information System (SINAN; *Sistema de Informação de Agravos de Notificação*) [12]. Because TB treatment is exclusively accessed via Brazil’s public healthcare system, TB case notifications are considered an accurate proxy for treatment initiation [10]. Case notifications were spatially referenced based on the recorded municipality of residence. In cases where municipality of residence was missing or coded incorrectly, municipality of treatment was used as a proxy (n = 202, 0.07%). We excluded cases that did not represent new diagnoses among living individuals (individuals who had received a misdiagnosis of TB (n = 6,063, 2.2%), were continuing care after transferring from another clinic (n = 7,762, 2.8%), were continuing care after previously being considered lost to follow-up (n = 22,242, 8.0%), or who had received a diagnosis of TB postmortem (n = 1,974, 0.7%)). In addition, we excluded cases where the municipality of residence had no neighboring municipalities (the island municipalities Ilhabela, SP and Fernando de Noronha, PE; n = 24, 0.009%). All case notifications had a recorded treatment outcome. Duplicate notifications are systematically removed from the database, and we did not detect any remaining duplicates in the analyzed dataset.

We accessed tuberculosis mortality data from 2016 to 2018 from the Brazilian Mortality Information System (SIM; *Sistem de Informação de Mortalidade*) [13]. We considered an individual to have died while actively infected with TB if at least one International Classification of Disease (ICD-10) code related to tuberculosis was listed as a primary or secondary cause of death [14]. We considered ICD-10 codes A15.0–A19.9, B20.0, K67.3, K93.0, M49.0, N74.0–N74.1, P37.0, U84.3; this list includes the code for TB/HIV co-infection. Deaths were spatially referenced based on municipality of residence. In addition, we used a linkage of the SIM and SINAN systems [15] to estimate the probability of death for case notifications with “death” or “loss to follow-up” as their reported treatment outcome.

Finally, we collated municipal-level data on sociodemographic characteristics (Table 1). These variables were chosen based on their expected relationship to TB burden [16] or the completeness of case detection.

**Table 1:** Model inputs.

Variable	Description	Data Source	Year(s) Used
**TB Case & Death Data**			
**Case Notifications**	Number of TB cases	SINAN-TB [12]	2016–18
**TB Mortality**	Number of deaths with a TB-related ICD-10 code as a primary or secondary cause	SIM [13]	2016–18
**TB Deaths after Notification**	The fraction of cases in SINAN-TB with “Death’ as an outcome that can be linked to a death in SIM occurring within 365 days of the case notification.	Bartholomay et al. [15]	2015–2016
**Treatment Outcome is “Death”**	Fraction of notified TB cases with a known outcome where “death” is the outcome	SINAN-TB [12]	2016–18
**Treatment Outcome is “Loss to Follow Up”**	Fraction of notified TB cases with a known outcome where “loss to follow-up” is the outcome	SINAN-TB [12]	2016–18
**Poorly Defined Cause of Death**	Percentage of deaths with a primary cause of death listed as an ICD-10 code considered "poorly defined" (ICD-10 codes R0-R99)	Health Informatics, Brazilian Ministry of Health (DATASUS) [13]	2016–18
**SIM Coverage**	Correction factor for under-reporting of all deaths in the mortality system (SIM)	Brazilian Institute of Geography and Statistics (IBGE) [17]	2018
**Population**	Population estimates by municipality or state	IBGE [17]	2016–18
**Sociodemographic Variables**			
Household Crowding^†^	Percentage of households with more than two people per bedroom	Atlas of Human Development in Brazil [18]	2010
Subnormal Agglomerations^†^	Percentage of population living in a structure classified as a "subnormal," including favelas and homes without access to running water or electricity	IBGE [17]	2010
Poverty^†^	Percentage of individuals earning R$255 (approximately $68) or less each month	Atlas of Human Development in Brazil [18]	2010
**Prison**	Indicates whether a municipality has a prison (binary) in any year included in the study period.	Brazilian Ministry of Justice and Public Security [19]	2016–18
**Public Hospital Beds**	Number of public hospital beds, per capita	DATASUS [20]	2016–18
**Primary Care Access**	Number of Family Health Teams per 4,000 people, by territory and year. One team per 4,000 population represents target coverage level; some municipalities surpass this coverage level.	DATASUS [20]	2016–18
**GDP Per Capita**	Economic value of goods produced	IBGE [17]	2016–18

† For the five municipalities created after the 2010 census, data from the municipality to which the territory previously belonged were used.

### Model description

We specified Poisson likelihood functions for total SINAN case notification data and SIM mortality data over the period 2016–2018.


$$\begin{array}{l} Case\;Notifications_{i}∼Poisson(\gamma_{i}∙\alpha_{i}∙\beta_{i})\\ TB\;Mortality_{i}∼Poisson(\gamma_{i}∙\alpha_{i}∙[\left(\beta_{i}∙\delta_{i})+(\left(1-\beta_{i})∙(1-\mu \right)\right)]∙b_{h_{i}}∙\varepsilon_{i}) \end{array}$$

For municipality *i*, where *δ*_*i*_ represents population size, α_*i*_ represents the modeled TB incidence rate, *β*_*i*_ represents the modeled fraction of cases treated, *δ*_*i*_ represents the probability of death during treatment, *μ* represents the probability of surviving the disease episode without treatment, *b*_*h*_ represents the fraction to total deaths recorded in SIM (calculated for the state (*h*) in which the municipality is located), and *ε*_*i*_ represents an adjustment for misreporting of TB deaths in the SIM database.

We followed the implementation of the modified Besag-York-Mollie (BYM2) model described by Morris et al. [21–23] to incorporate the spatial structure of municipalities in our model. We selected the BYM2 model because it does not presume spatial autocorrelation; the model includes a mixing parameter which distributes random variance in model estimates across spatial and non-spatial effects, making it an appropriate choice when the presence of spatial autocorrelation is uncertain [24]. We specified exponential and inverse logit functions for incidence (α_i_) and fraction treated (β_i_), respectively:

$$\begin{array}{l} \alpha_{i}=exp(\pi_{0}+{\pi_{1}}_{i}+X_{i}^{T}\pi )\\ \beta_{i}=logit^{-1}(\omega_{0}+{\omega_{1}}_{i}+Z_{i}^{T}\omega ) \end{array}$$

For municipality *i*, where π_0_ and ω_0_ are constants; *X*_*i*_ and Z_i_ are vectors of municipal-level covariates; and π and ω are the associated vectors of regression coefficients. The vector *X*_*i*_ includes the covariates *percentage of crowded househol*ds, *percentage of municipality classified as a favela (slum) or other subnormal housing*, *percentage of individuals in poverty*, whether there is a *prison* in the municipality, *number of public hospital bed*s *per capita*, *primary healthcare access*, and *average GDP per capita*. The vector *Z*_*i*_ includes the *covariates percentage of individuals in poverty*, whether there is a *prison* in the municipality, *number of public hospital beds per capita*, *primary healthcare access*, and *average GDP per capita*. Apart from individuals recorded as having been misdiagnosed, we assumed that all notified cases represent individuals who truly had TB. Similarly, we did not consider false-positive diagnosis among SIM TB death data. Finally, π_1i_ and ω_1i_ are the combined random and spatial effects of the BYM2 model:

$$\begin{array}{l} \pi_{1i}=(\sqrt{1-\rho_{\pi }}∙\theta_{\pi ,i}+\sqrt{\frac{\rho_{\pi }}{S}}∙\phi_{\pi ,i})∙\sigma_{\pi }\\ \omega_{1i}=(\sqrt{1-\rho_{\omega }}∙\theta_{\omega ,i}+\sqrt{\frac{\rho_{\omega }}{S}}∙\phi_{\omega ,i})∙\sigma_{\omega } \end{array}$$

For municipality *i*, where *φ*_*i*_ represents an intrinsic conditional auto-regressive spatial component, *θ*_*i*_ is a non-spatial random component, *ρ* models the proportion of variance from the spatially structured effect, and *S* is a scaling factor computed from the spatial adjacency matrix. The scaling factor is chosen such that the Var(φ_*i*_) ≈ 1; additionally, the prior on θ is fixed such that Var(*θ*_*i*_) ≈ 1, making σ the overall standard deviation of the combined random effects component [21–23].

In addition, we estimated the probability of death among individuals who initiated treatment:

$${Pr(death|treatment)}_{i}=\delta_{i}=\zeta_{i}*\lambda +\iota_{i}*\eta$$

For municipality *i*, where *ζ*_*i*_ is the probability that death is listed as the treatment outcome, *ι*_*i*_ is the probability that loss to follow-up is listed as the treatment outcome, λ is that probability that an individual with treatment outcome of death will appear in the death record, and *η* is the probability that an individual whose treatment outcome was lost to follow up will appear in the death record. We determined the means of the prior distributions for *ζ*_*i*_ and *ι*_*i*_ from state-level data, dividing the number of outcomes listed as death and lost to follow-up, respectively, by the number of treatment notifications with a definitive treatment outcome (cure, death, loss to follow-up, or treatment failure). We calculated the means of the prior distributions for λ and *η* based on a linkage of SINAN and SIM [15]. We calculated the fraction of individuals with a treatment outcome of “death” or “loss to follow-up” (respectively) linked to a death record in SIM within 365 days of their case notification. Because 2016 was the last year for which death data were available in the linked dataset, we restricted this analysis to case notifications from 2015.

Finally, we estimated the systematic underreporting of TB as a cause of death:

$$Death\;Adjustment_{i}=\varepsilon_{i}=logit^{-1}(\kappa_{1}+\kappa_{2i}*\sigma_{\kappa }+\kappa_{3}x_{i})$$

For municipality *i*, where *κ*_1_ is a constant, *κ*_2_ is a municipal-level random effect, σ_*κ*_ is the standard deviation, *x*_*i*_ is the percentage of deaths in SIM attributed to a poorly-defined cause, and *κ*_3_ is the associated regression coefficient (based on values elicited through an expert opinion survey [10]). We assumed that underreporting of TB as a cause of death is not a spatially dependent process.

### Estimation and Implementation

There is substantial uncertainty around true values for several model parameters. We used a Bayesian approach to represent and propagate this uncertainty through the analysis, utilizing prior probability distributions to describe plausible ranges for model parameters (S1 Table). When fitting the model likelihood functions to data, we used a three years of input municipality data. Summing cases and deaths over a three-year period decreased the stochastic uncertainty associated with low case counts and reduced the number of municipalities with no reported TB deaths in the study period, improving inference for these municipalities.

The model was implemented in stan [25] using the rstan [26] package. We ran 6000 iterations on four chains, keeping 500 iterations after warm-up and thinning by a factor of two for a total of 1000 posterior draws. Running on four-chains in parallel using the Yale Center for Research Computing’s high-performance computing cluster took approximately 8.5 hours, based on the reported elapsed time. The mean $\hat{R}$ was 1.005 and there were no diagnostic warnings. We calculated point estimates as the mean of the posterior draws. We calculated equal-tailed 95% posterior intervals using the 2.5^th^ and 97.5^th^ percentiles of these distributions. To calculate state- and national-level results we summed estimates across municipalities for each posterior draw before calculating point estimates and intervals. Parameter posterior distributions can be found in S2 Table. Input data and model code are available at github.com/mel-hc/TB_saie.

## Results

Over the period 2016–2018, there were 239,369 individuals who initiated treatment for a newly diagnosed case of TB. Nationally, we observed 38.5 notified TB cases per 100,000 person-years over the three-year study period. In this same period, there were 59,183 deaths where TB was listed on the death certificate (as the underlying or contributory cause), corresponding to 3.5 deaths per 100,000 person-years.

### TB incidence

**Fig 1:**
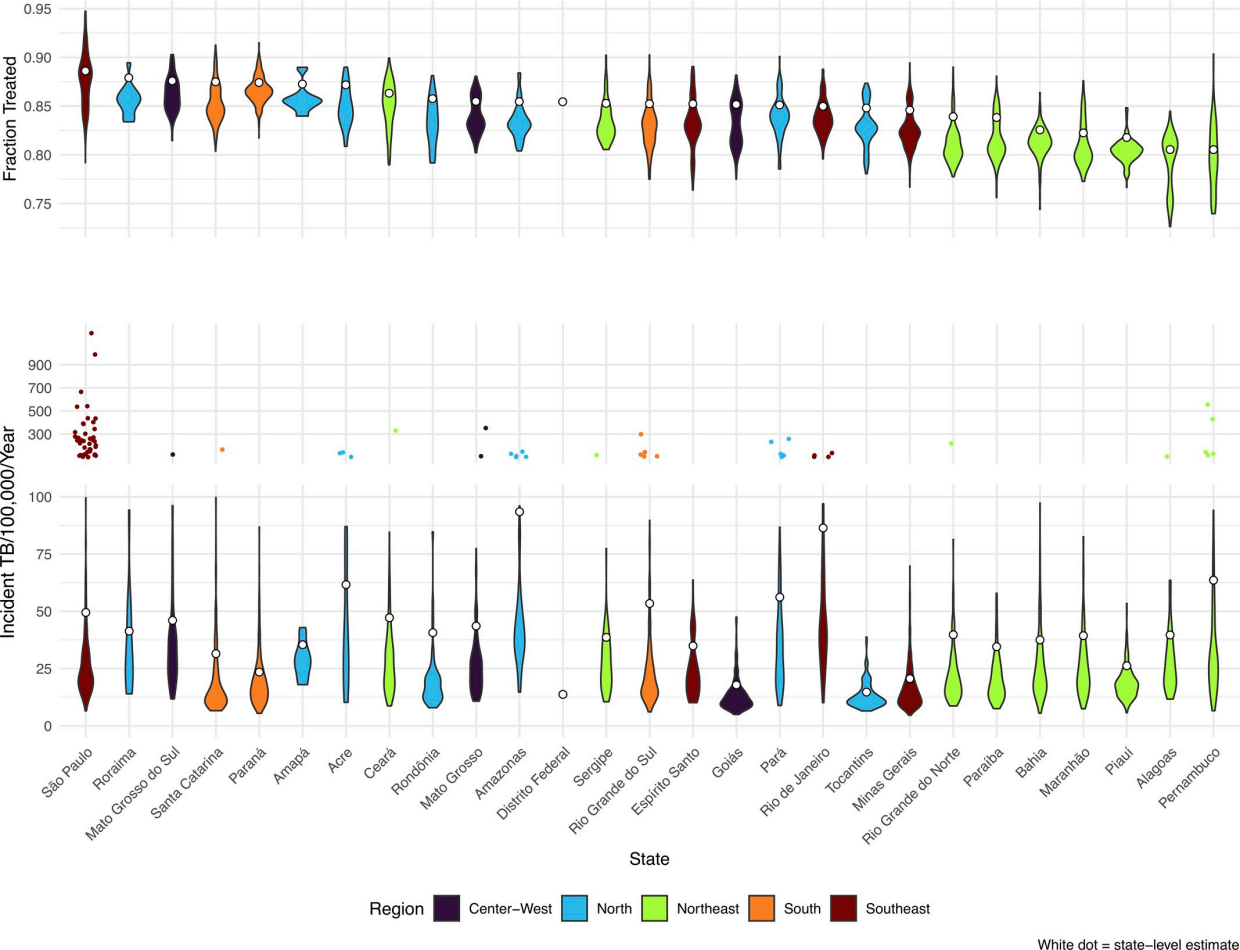
Violin plots of fraction treated (top) and incident TB (bottom). White dots represent the population-weighted state averages.

We estimated a national incidence rate of 44.8 (95% credible interval: 43.3, 46.8) per 100,000 person-years, for the three-year study period. The TB incidence rate varied substantially across and within states (Fig 1). Average state-level incidence rate ranged from 13.8 (12.7, 15.0) per 100,000 person years in the Federal District to 93.4 (88.8, 98.9) in Amazonas.

**Fig 2:**
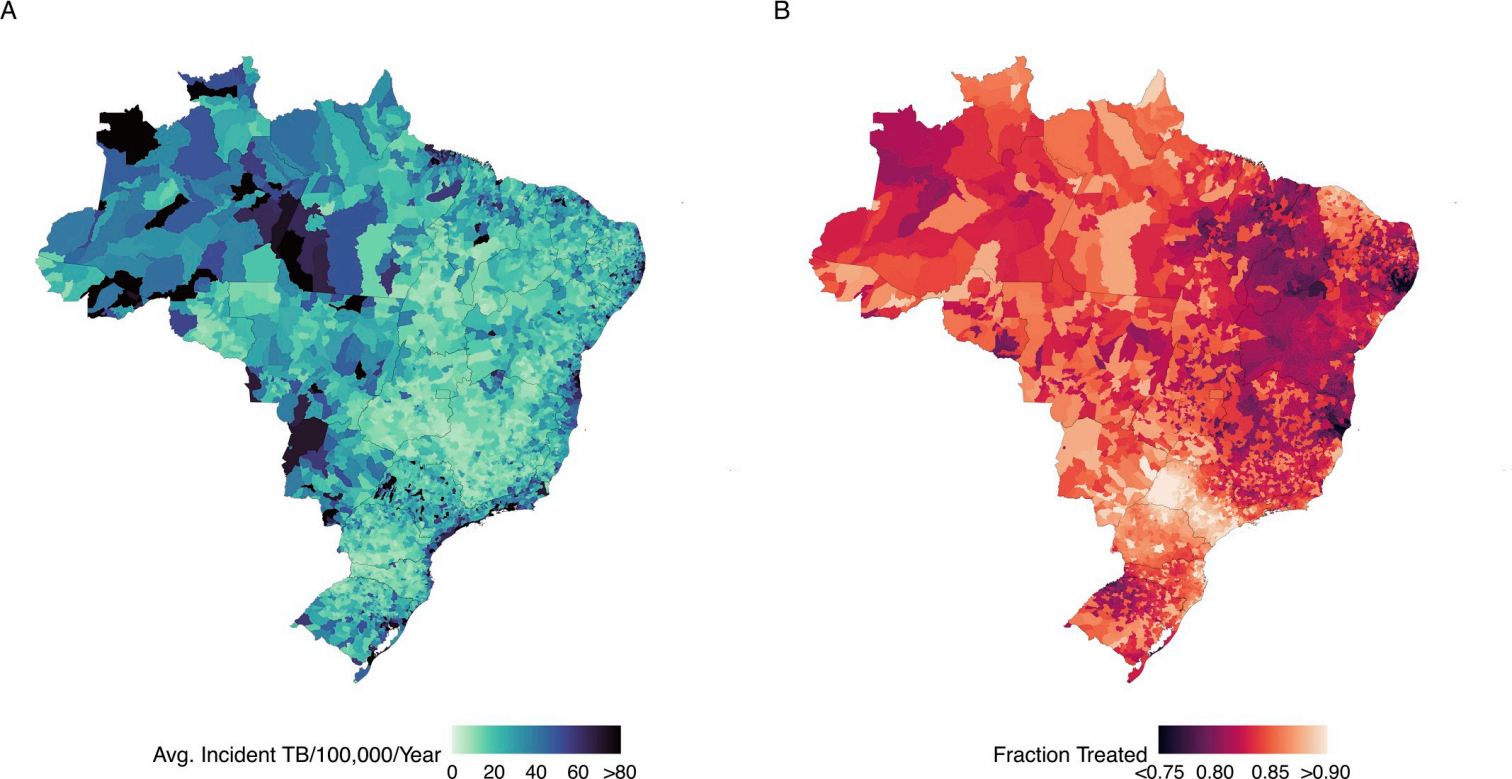
Map of municipal estimates of (A) incident TB per 100,000 population per year and (B) the fraction of individuals with incident TB receiving treatment. Shapefiles were downloaded using the geobr package for the R programming language, which sources shapefiles from IBGE: https://www.ibge.gov.br/en/geosciences/territorial-organization/territorial-meshes/.

The median municipal incidence rate was 19.3 per 100,000 and the estimated incident TB rate ranged from 4.6 to 1172 per 100,000 per year (S1A Fig). The majority of incident TB cases were concentrated in a small number of municipalities. Over half of TB incidence in each state was attributed to 1.1% to 6.7% of municipalities within that state. In Amazonas, 72.5% (71.2%, 73.7%) of incident TB occurred among individuals living in Manaus, and in Rio de Janeiro State, 52.2% (51.2%, 53.3%) of incident TB occurred among individuals living in Rio de Janeiro municipality. Nationally, 1% of municipalities accounted for 50.0% (49.7%, 50.4%) of incident TB. Because Brazil’s population is highly concentrated in urban municipalities, we expect a small number of municipalities to account for a large share of incident TB. While these larger municipalities share several features that promote TB transmission (high population density, populations living in subnormal agglomerations), we found a relatively weak correlation between municipal population size and TB incidence rate (Pearson correlation coefficient (ρ) = 0.09, p < 0.001). A map of incident TB rates, by municipality, can be found in Fig 2A.

A small number of municipalities (n = 39) had a mean estimated incident TB rate in excess of 150 per 100,000 inhabitants per year. Most of these municipalities had a large number of notified cases (average notified cases = 217) relative to their small population (average population = 28,855). Additionally, 95% of these municipalities (n = 37) contained a prison, and for these municipalities the incarcerated population was large relative to total population (median 175 incarcerated individuals to 1000 inhabitants) (S3 Table). Across all municipalities, the presence of a prison was a significant predictor of elevated TB incidence rate, corresponding to a 1.52 (1.46, 1.59) increase in the rate ratio of TB incidence, conditional on socioeconomic factors and healthcare quality. Collectively, municipalities with prisons had a higher TB incidence rate (51.9 [50.2, 54.2]) than municipalities without prisons (33.5 [32.2, 35.3]), p < 0.001). In municipalities with prisons, TB incidence rates correlate strongly with the number of incarcerated individuals per 1000 residents (Pearson correlation coefficient (ρ) = 0.87, p < 0.001). Municipalities with more than 10 incarcerated individuals to 1000 inhabitants had a median TB incidence rate of 67.7 (range: 9.2, 1172) per 100,000.

### Fraction of incident TB receiving treatment

We estimated that the average fraction of incident TB cases receiving treatment in Brazil was 0.86 (0.82, 0.89). As with estimates of incident TB, the fraction of treated cases varied by state, from 0.81 (0.75, 0.85) in Alagoas, 0.89 (0.86, 0.92) in São Paulo. Across municipalities, the estimated fraction treated ranged from 0.73 to 0.95 (S1B Fig). Areas where the fraction of TB cases that receive treatment is low are generally concentrated in the Northeast region of the country (Fig 2B).

### Rate of untreated TB

**Fig 3:**
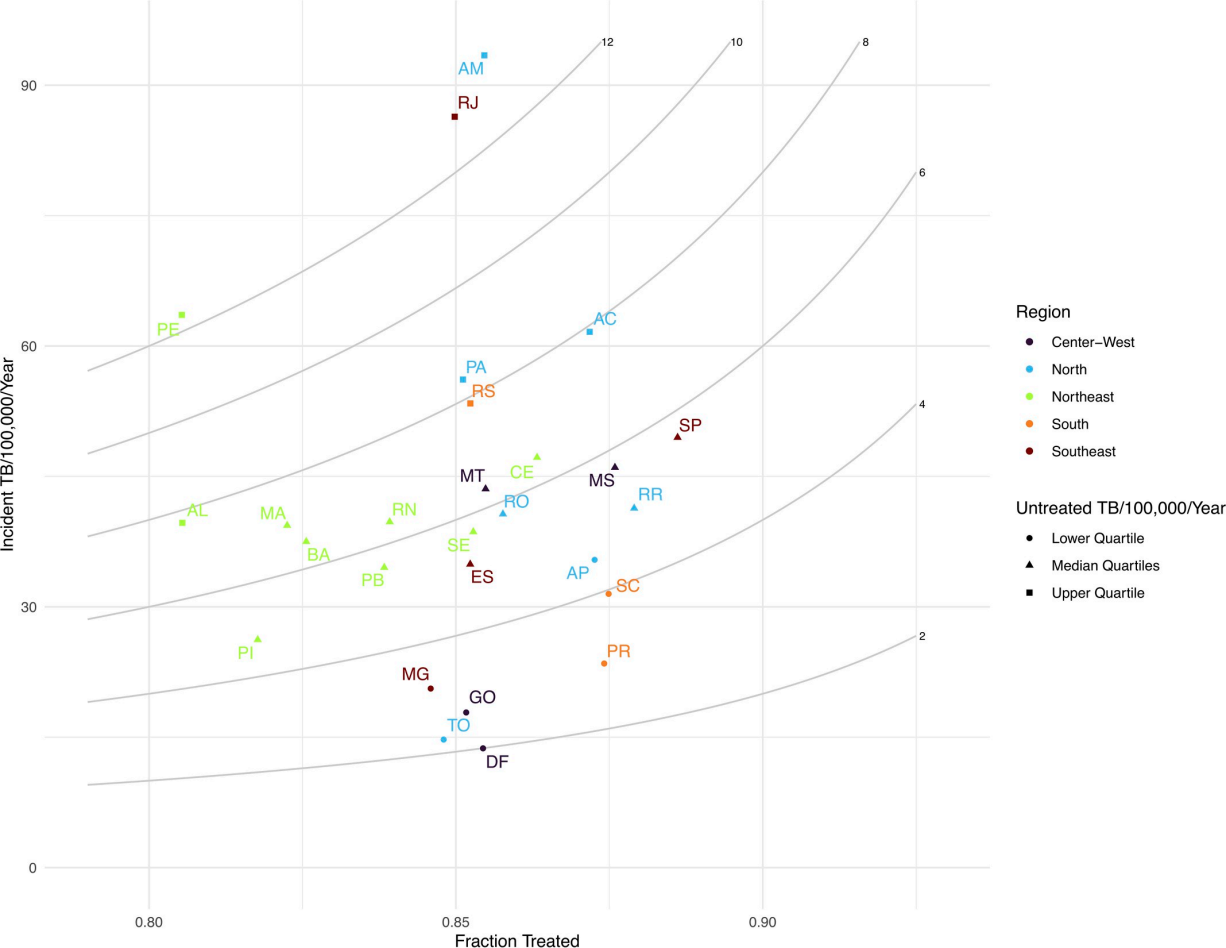
State-level estimates of the incident TB rate per 100,000 population per year (y-axis) and average fraction treated per year (x-axis), with isopleths of untreated TB per 100,000 population per year (grey curved lines).

We estimate the rate of untreated tuberculosis as the product of the incident TB rate and 1 minus the fraction treated. Nationally, there were an average 13,049 (9941, 17247) individuals with incident TB that did not receive treatment each year (6.3 [4.8, 8.3] per 100,000 person-years). Rates of untreated TB varied substantially across states, from 2.0 (1.3, 3.0) per 100,000 in the Federal District to 13.1 (8.9, 18.8) in Amazonas (Fig 3).

**Fig 4:**
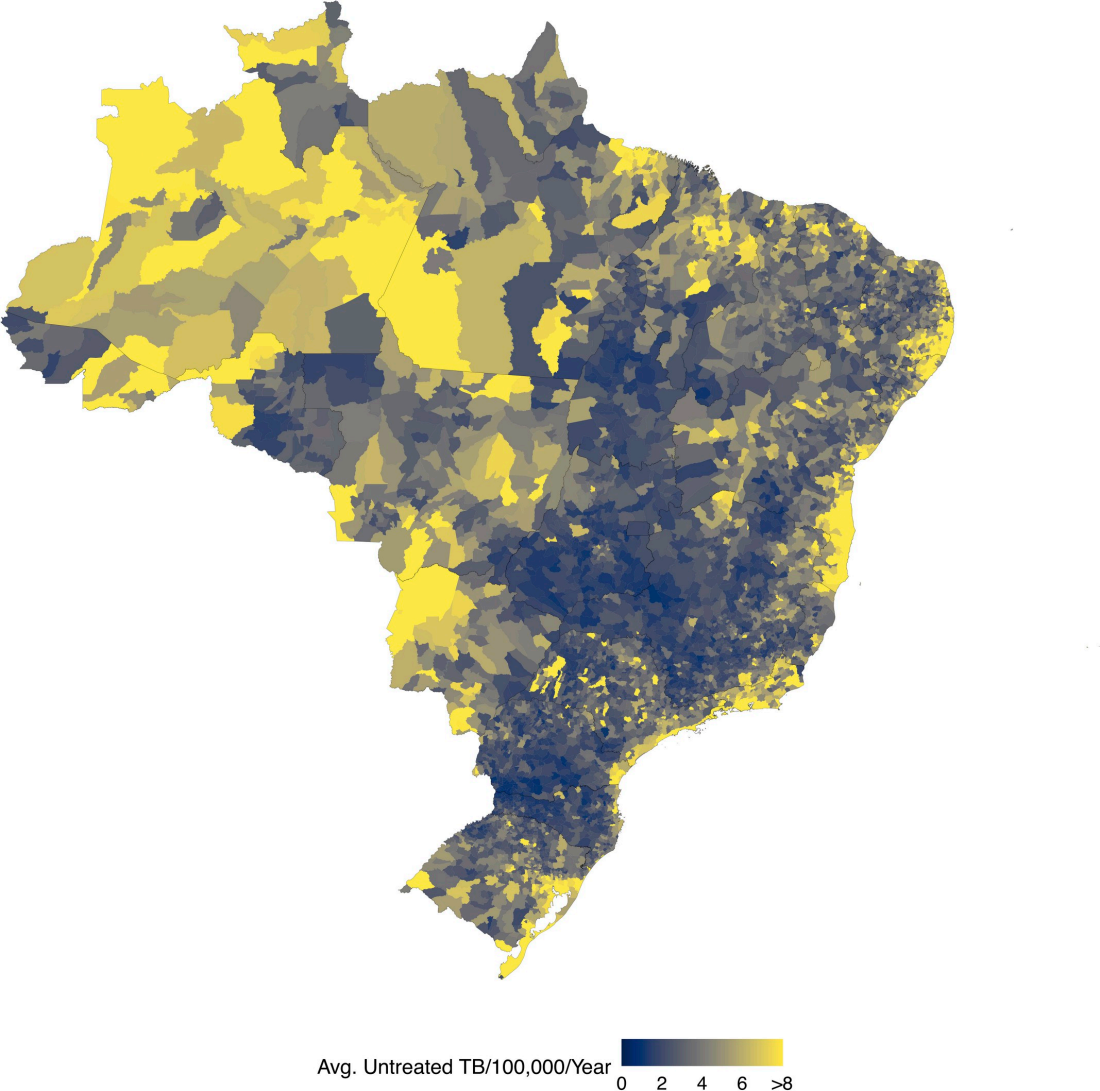
Map of municipal estimates of untreated incident TB per 100,000 population per year. Shapefiles were downloaded using the geobr package for the R programming language, which sources shapefiles from IBGE: https://www.ibge.gov.br/en/geosciences/territorial-organization/territorial-meshes/.

We estimated high rates of untreated TB throughout Brazil, but found that these areas were generally concentrated in urban coastal municipalities and in the North region (Fig 4). Rio de Janeiro municipality had the largest number of untreated TB of any municipality, with an average 1031 (664, 1469) untreated TB cases each year. Collectively, the 65 municipalities with the highest number of untreated individuals accounted for 50.2% (47.8%, 52.7%) of untreated incident TB in Brazil (S4 Table). A 1% increase in the fraction treated across these 65 municipalities would result in 477 (462, 501) fewer untreated individuals each year; a 1% increase in fraction treated across all municipalities would result in 926 (897, 970) fewer untreated individuals each year.

### Model sensitivity to prior distributions

We conducted sensitivity analyses to understand the impact of the choice of prior distribution for the probability of survival without treatment (an input estimated based on expert opinion). First, we re-fit the model with weakly informative priors and found that the posterior distribution was sensitive to the choice of model prior. We also found that the posterior probability of survival without treatment correlates strongly with national estimates of the TB incidence rate (Pearson correlation coefficient (ρ) = 0.89, p < 0.001) and fraction treated (Pearson correlation coefficient (ρ) = -0.90, p <0.001) (S2 Fig). However, the relative rank of municipal incidence rate and treatment coverage estimates when the model was run with strong priors around extreme values (0.3, 0.7) of survival without treatment were very strongly correlated (Spearman’s rank correlation coefficient (ρ) = 0.99, p < 0.001 and (ρ) = 0.98, p < 0.001, respectively).

## Discussion

We present a spatially-explicit Bayesian model to estimate rates of TB incidence and treatment initiation. We demonstrate the feasibility of this approach for generating small area disease burden estimates with routinely-collected data on case notifications and deaths. Applying this approach to data from Brazil, we found substantial subnational variation in TB epidemiology. Consequently, a small number of municipalities were responsible for the majority of incident TB in Brazil. Additionally, we found that the presence of prison in a municipality was associated with higher TB rates, a finding which is supported by previous work on TB incidence in Brazilian prisons [27] and their surrounding communities [28]. We present a metric of untreated TB which can be used to identify municipalities where a large number of incident TB cases did not initiate treatment (Fig 3, S4 Table). Improving case detection and treatment initiation rates in these municipalities could be an effective strategy for reducing the burden of TB in Brazil. Finally, we aggregated municipal-level results to the state-level and found that our point estimates for incidence and fraction treated generally fell within the uncertainty bounds of previously published state-level estimates [10], though with lower estimates of the fraction treated (S3 Fig). Aggregating to the national level, our estimates of incident TB per 100,000 person years (44.8 [43.4, 46.8]) and fraction treated (0.86 [0.82, 0.89]) were consistent with estimates produced by the WHO (45 [39, 52], and 0.87 [0.75, 1.00], respectively) [1].

This approach is not the first that has been proposed to generate subnational TB burden estimates. One approach that has been used in other large high TB burden countries is the SUBsET method [7,8]. As applied in Indonesia, this method starts from WHO-estimated TB incidence for the country, then decomposes this total into district-level values based on predictors defined through expert elicitation (population size, urbanization, socio-economic indicators). Case detection rates are then calculated as the ratio of notifications to estimated incidence. While this method leverages local knowledge on epidemiological drivers, it excludes TB notifications and death data from the incidence estimation, which may provide valuable additional signals of disease burden. In another published study, province-level mathematical models were constructed for South Africa to estimate both disease burden estimates and investigate policy scenarios [9]. While the use of fully-developed policy models allowed this study to directly compare control options in each province, the inference approach was not designed to identify differences in case detection between provinces, and instead a common case detection rate was applied in all locations based on the national WHO estimate. A recent study in Ethiopia employed a Hidden Markov Model and spatiotemporal smoothing to link case notifications to unobserved TB incidence. This approach allowed for local differences in case detection rates, with these differences identified primarily through spatiotemporal random effects and the presence or absence of a health facility [29]. Other studies that have provided high granularity burden estimates have smoothed case notifications directly, implicitly assuming perfect case detection rates [6]. Finally, several approaches have been developed to leverage TB prevalence survey data to produce subnational TB burden estimates (applied in Pakistan [30] and Bangladesh [31]). While prevalence survey data represent a valuable additional source of information on the distribution of TB, these surveys are expensive to undertake and are not available for all countries.

Although the approach described here potentially resolves many of these limitations, this study has several limitations. First, the model is designed to correct for the misattribution of TB deaths to other causes, but this adjustment may be insufficient in some cases, leading to underestimates of incident TB rates in municipalities with exceptionally poor death records. Secondly, we found that the posterior distribution of the probability of survival without treatment was dependent on the choice of prior distribution, and model outcomes were sensitive to this parameter. The probability of death without treatment in the antibiotic era is not easily observable; our choice of prior distribution represents our best possible estimate. However, if the probability of survival without treatment were lower than our estimate, the model would underestimate the incident TB rate and overestimate the fraction treated (S2 Fig). Thirdly, our analytic strategy did not consider the possibility of overdiagnosis of TB. These false-positive diagnoses are an acknowledged risk of TB diagnostic algorithms, particularly when TB is diagnosed based on clinical criteria [32]. This could produce biased estimates of the fraction treated by overestimating treatment initiations and treatment fatality rates. Over the study period, we found that 87% of notified cases in Brazil were tested with highly-specific bacteriological tests (microscopy, culture, or molecular (GeneXpert) tests). Among notified cases without one of these tests, 72% had x-ray findings indicative of TB. These figures suggest that overdiagnosis is a relatively minor feature of TB diagnosis in Brazil, and unlikely to meaningfully affect our modelled estimates. Our analysis also did not include primary abandonment (diagnosed individuals who do not initiate treatment or who default within the first 30 days), however programmatic data suggest these individuals represent less than 1% of all notifications.

Finally, this analysis did not consider some risk factors for TB mortality that may have improved inference. The most important of these is HIV, which substantially increases mortality rates for individuals with TB disease [33,34]. If individuals who are co-infected with TB and HIV are more likely to die from TB in the absence of treatment, the municipalities with high TB-HIV coinfection rates could have upwardly biased incidence estimates. Conversely, if TB-HIV deaths are more likely to be misclassified as other categories of HIV/AIDS related deaths, this would mean that incidence estimates for high TB-HIV municipalities would be biased downward. The net effect of these two potential biases is unclear. Although HIV surveillance in Brazil is believed to be reasonably complete [35], HIV testing is not consistently performed with TB diagnosis (17.5% not performed or missing), making it difficult to infer rates of TB-HIV co-infection for this analysis. It may be necessary to extend this analytic approach to explicitly account for TB-HIV in order to apply it in high HIV prevalence settings.

The approach presented here can be a valuable tool enabling countries to better understand TB burden on a fine spatial scale. This approach uses routinely-collected data on case notifications and deaths, making it applicable to settings where the number of individuals receiving TB treatment is known and quality cause-specific death records are available. In settings where a large fraction of areas has no reported cases or deaths in a given year, aggregating over multiple years can reduce stochastic uncertainty in model results. The modified Besag-York-Mollié spatial and random effect terms can also be extended to account for non-contiguous areas. Furthermore, because the modified Besag-York-Mollié approach has been shown to be unbiased in applications with no spatial autocorrelation [24], the model does not need to be reparametrized for settings where the spatial autocorrelation in TB burden may be small or negligible.

Small area incidence estimates generated from this approach can help local health agencies tailor programmatic improvements to their specific epidemiological situations. Targeting improvements in TB case finding in municipalities with high rates of untreated TB could result in large increases in the number of infected individuals initiating treatment and ultimately decrease TB incidence and mortality.

## Supporting information

S1 Fig Histogram of municipal estimates of (A) incident TB per 100,000 population per year and (B) the fraction of individuals with incident TB receiving treatment.
(TIFF)

S2 Fig Correlation between the probability of survival without treatment (x-axis), the national incidence estimate (y-axis) and the fraction of TB cases initiating treatment (color gradient). (TIFF)

S3 Fig Comparison of state-level estimates of (A) incident TB per 100,000 population per year and (B) the fraction of individuals with incident TB receiving treatment estimated with the spatial-mechanistic model described in this manuscript (red) and previously published state-level estimates (blue).
(TIFF)

S1 Table Model parameters. (DOCX)

S2 Table Parameter posterior distributions. (DOCX)

S3 Table Municipalities with the highest TB incidence rates. (DOCX)

S4 Table Municipalities with the highest number of individuals with untreated TB. (DOCX)

## References

[1] Global Tuberculosis Report. Geneva: World Health Organization; 2019.

[2] Implementing the end TB Strategy: The Essentials. Geneva: World Health Organization; 2015.

[3] Burke RM, Nliwasa M, Feasey HRA, Chaisson LH, Golub JE, Naufal F, et al. Community-based active case-finding interventions for tuberculosis: a systematic review. The Lancet Public Health. 2021;6(5):e283–e99. doi: 10.1016/S2468-2667(21)00033-5 33765456 PMC8082281

[4] Marks GB, Nguyen NV, Nguyen PTB, Nguyen TA, Nguyen HB, Tran KH, et al. Community-wide Screening for Tuberculosis in a High-Prevalence Setting. N Engl J Med. 2019;381(14):1347–57. doi: 10.1056/NEJMoa1902129 31577876

[5] Dowdy DW, Golub JE, Chaisson RE, Saraceni V. Heterogeneity in tuberculosis transmission and the role of geographic hotspots in propagating epidemics. Proc Natl Acad Sci U S A. 2012;109(24):9557–62. doi: 10.1073/pnas.1203517109 22645356 PMC3386125

[6] Martins-Melo FR, Bezerra JMT, Barbosa DS, Carneiro M, Andrade KB, Ribeiro ALP, et al. The burden of tuberculosis and attributable risk factors in Brazil, 1990–2017: results from the Global Burden of Disease Study 2017. Popul Health Metr. 2020;18(Suppl 1):10. doi: 10.1186/s12963-020-00203-6 32993691 PMC7526097

[7] Parwati CG, Farid MN, Nasution HS, Basri C, Lolong D, Gebhard A, et al. Estimation of subnational tuberculosis burden: generation and application of a new tool in Indonesia. Int J Tuberc Lung Dis. 2020;24(2):250–7. doi: 10.5588/ijtld.19.0139 32127111

[8] Mulder C, Nkiligi E, Kondo Z, Scholten JN. What to look for when using SUBsET for subnational TB incidence estimates. Int J Tuberc Lung Dis. 2020;24(9):983–4. doi: 10.5588/ijtld.20.0188 33156772

[9] Doherty T, Hippner P, Sumner T, Houben RMGJ, Cardenas V, Vassall A, et al. Application of provincial data in mathematical modelling to inform sub-national tuberculosis program decision-making in South Africa. Plos One. 2019;14(1). 10.1371/journal.pone.0209320 PMC6347133 30682028

[10] Chitwood MH, Pelissari DM, Drummond Marques da Silva G, Bartholomay P, Rocha MS, Sanchez M, et al. Bayesian evidence synthesis to estimate subnational TB incidence: An application in Brazil. Epidemics. 2021;35. doi: 10.1016/j.epidem.2021.100443 33676092 PMC8252152

[11] Chitwood MH, Pelissari DM, Marques da Silva GD, Bartholomay P, Rocha MS, Arakaki-Sanchez D, et al. Trends in Untreated Tuberculosis in Large Municipalities, Brazil, 2008–2017. Emerg Infect Dis. 2021;27(3):957–60. doi: 10.3201/eid2703.204094 33622464 PMC7920690

[12] Brasil Ministério da Saúde. Secretaria de Vigilância em Saúde. Departamento de Vigilância Epidemiológica. Sistema de Informação de Agravos de Notificação–Sinan. Available from: http://tabnet.datasus.gov.br/cgi/menu_tabnet_php.htm [Accessed June 1 2021].

[13] Brasil Ministério da Saúde. Secretaria de Vigilância em Saúde. Eventos Vitais–Sistema de Informação sobre Mortalidade (SIM). Available from: http://tabnet.datasus.gov.br/cgi/deftohtm.exe?sim/cnv/obt10uf.def [Accessed June 1 2021].

[14] International Statistical Classification of Diseases and Related Health Problems 10th Revision 2016.

[15] Bartholomay P, Oliveira GP, Pinheiro RS, Vasconcelos AM. [Improved quality of tuberculosis data using record linkage.]. Cad Saude Publica. 2014;30(11):2459–70. 25493999 10.1590/0102-311x00116313

[16] Harling G, Castro MC. A spatial analysis of social and economic determinants of tuberculosis in Brazil. Health Place. 2014;25:56–67. doi: 10.1016/j.healthplace.2013.10.008 24269879

[17] Sistema Ibge de Recuperação Automática–SIDRA [Internet]. Instituto Brasileiro de Geografia e Estatística—IBGE. Available from: https://sidra.ibge.gov.br/home/pimpfbr/brasil [Accessed June 1 2021].

[18] Atlas of Human Development in Brazil [Internet]. United Nations Development Programme. Available from: http://www.atlasbrasil.org.br/. [Accessed June 1 2021].

[19] Ministério da Justiça e Segurança Pública. Departamento Nacional Penitenciário. Levantamento Nacional de Informações Penitenciárias: INFOPEN. Available from: https://dados.mj.gov.br/dataset/infopen-levantamento-nacional-de-informacoes-penitenciarias. [Accessed June 1 2021].

[20] Brasil, Ministério da Saúde. Banco de dados do Sistema Unico de Saúde-DATASUS Available from: http://www.datasus.gov.br. [Accessed June 1 2021].

[21] Morris M, Wheeler-Martin K, Simpson D, Mooney SJ, Gelman A, DiMaggio C. Bayesian hierarchical spatial models: Implementing the Besag York Mollie model in stan. Spat Spatiotemporal Epidemiol. 2019;31:100301. 31677766 10.1016/j.sste.2019.100301 PMC6830524

[22] Morris M. nb_data_funs.R [Source code]. Available from: https://github.com/stan-dev/example-models/blob/885bd18e93fd4b7b19290d8967064174bbe45156/knitr/car-iar-poisson/nb_data_funs.R. [Accessed February 15 2020].

[23] Morris M. bym2.stan [Source Code]. Available from: https://github.com/stan-dev/example-models/blob/885bd18e93fd4b7b19290d8967064174bbe45156/knitr/car-iar-poisson/bym2.stan [Accessed February 15 2020].

[24] Riebler A, Sorbye SH, Simpson D, Rue H. An intuitive Bayesian spatial model for disease mapping that accounts for scaling. Stat Methods Med Res. 2016;25(4):1145–65. doi: 10.1177/0962280216660421 27566770

[25] Stan Development Team. Stan Modeling Language Users Guide and Reference Manual. Version 2.27.0. 2019.

[26] Stan Development Team. RStan: the R interface to stan. 2018.

[27] Carbone Ada S, Paiao DS, Sgarbi RV, Lemos EF, Cazanti RF, Ota MM, et al. Active and latent tuberculosis in Brazilian correctional facilities: a cross-sectional study. BMC Infect Dis. 2015;15:24. doi: 10.1186/s12879-015-0764-8 25608746 PMC4307675

[28] Walter KS, dos Santos PCP, Gonçalves TO, da Silva BO, da Silva Santos A, de Cássia Leite A, et al. Genomic evidence for prisons as amplifiers of community tuberculosis epidemics 2021.

[29] Shaweno D, Trauer JM, Denholm JT, McBryde ES. A novel Bayesian geospatial method for estimating tuberculosis incidence reveals many missed TB cases in Ethiopia. BMC Infectious Diseases. 2017;17(1). doi: 10.1186/s12879-017-2759-0 28969585 PMC5625624

[30] Alba S, Rood E, Mecatti F, Ross JM, Dodd PJ, Chang S, et al. TB Hackathon: Development and Comparison of Five Models to Predict Subnational Tuberculosis Prevalence in Pakistan. Trop Med Infect Dis. 2022;7(1). doi: 10.3390/tropicalmed7010013 35051129 PMC8780063

[31] Allorant A, Biswas S, Ahmed S, Wiens KE, LeGrand KE, Janko MM, et al. Finding gaps in routine TB surveillance activities in Bangladesh. Int J Tuberc Lung Dis. 2022;26(4):356–62. doi: 10.5588/ijtld.21.0624 35351241 PMC8982646

[32] Houben R, Lalli M, Kranzer K, Menzies NA, Schumacher SG, Dowdy DW. What if They Don’t Have Tuberculosis? The Consequences and Trade-offs Involved in False-positive Diagnoses of Tuberculosis. Clin Infect Dis. 2019;68(1):150–6. doi: 10.1093/cid/ciy544 29982375 PMC6293007

[33] Schmaltz CA, Sant’Anna FM, Neves SC, Velasque L, Lourenço MC, Morgado MG, et al. Influence of HIV Infection on Mortality in a Cohort of Patients Treated for Tuberculosis in the Context of Wide Access to HAART, in Rio de Janeiro, Brazil. J Acquir Immune Defic Syndr. 2009;25(5):623–8. 10.1097/QAI.0b013e3181b31e56 19730270

[34] Sanchez M, Bartholomay P, Arakaki-Sanchez D, Enarson D, Bissell K, Barreira D, et al. Outcomes of TB treatment by HIV status in national recording systems in Brazil, 2003–2008. PLoS One. 2012;7(3):e33129. doi: 10.1371/journal.pone.0033129 22457738 PMC3310054

[35] Mangal TD, Pascom ARP, Vesga JF, Meireles MV, Benzaken AS, Hallett TB. Estimating HIV incidence from surveillance data indicates a second wave of infections in Brazil. Epidemics. 2019;27:77–85. doi: 10.1016/j.epidem.2019.02.002 30772250 PMC6543066

