# Supplementary figures and images for: A spatial-mechanistic model to estimate subnational tuberculosis burden with routinely collected data: An application in Brazilian municipalities
Source: PLOS Glob Public Health. 2022 Sep 21;2(9):e0000725. doi: 10.1371/journal.pgph.0000725 (PMC10021638; doi:10.1371/journal.pgph.0000725)

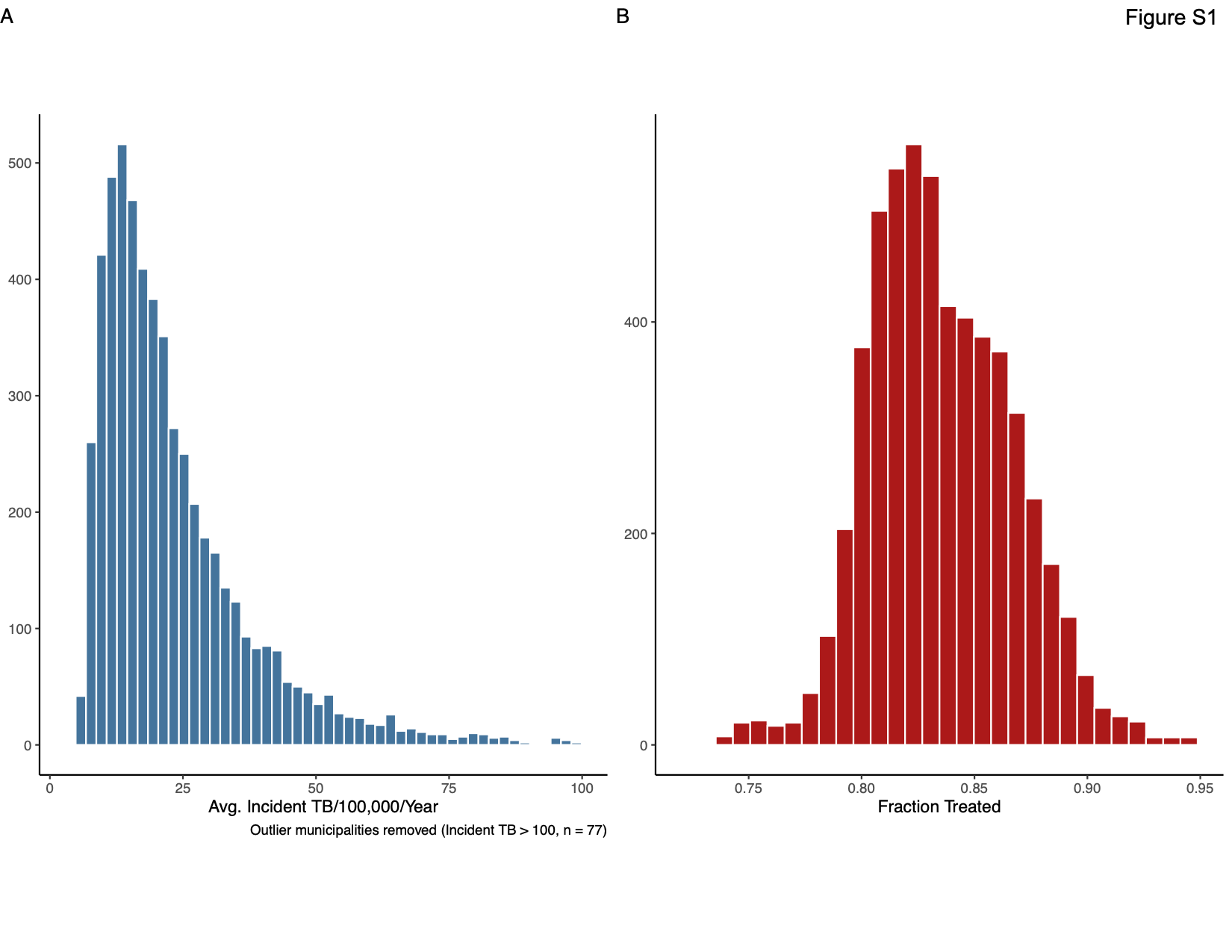

Supplement: S1 Fig — Histogram of municipal estimates of (A) incident TB per 100,000 population per year and (B) the fraction of individuals with incident TB receiving treatment. (TIFF) [file pgph.0000725.s001.tiff]

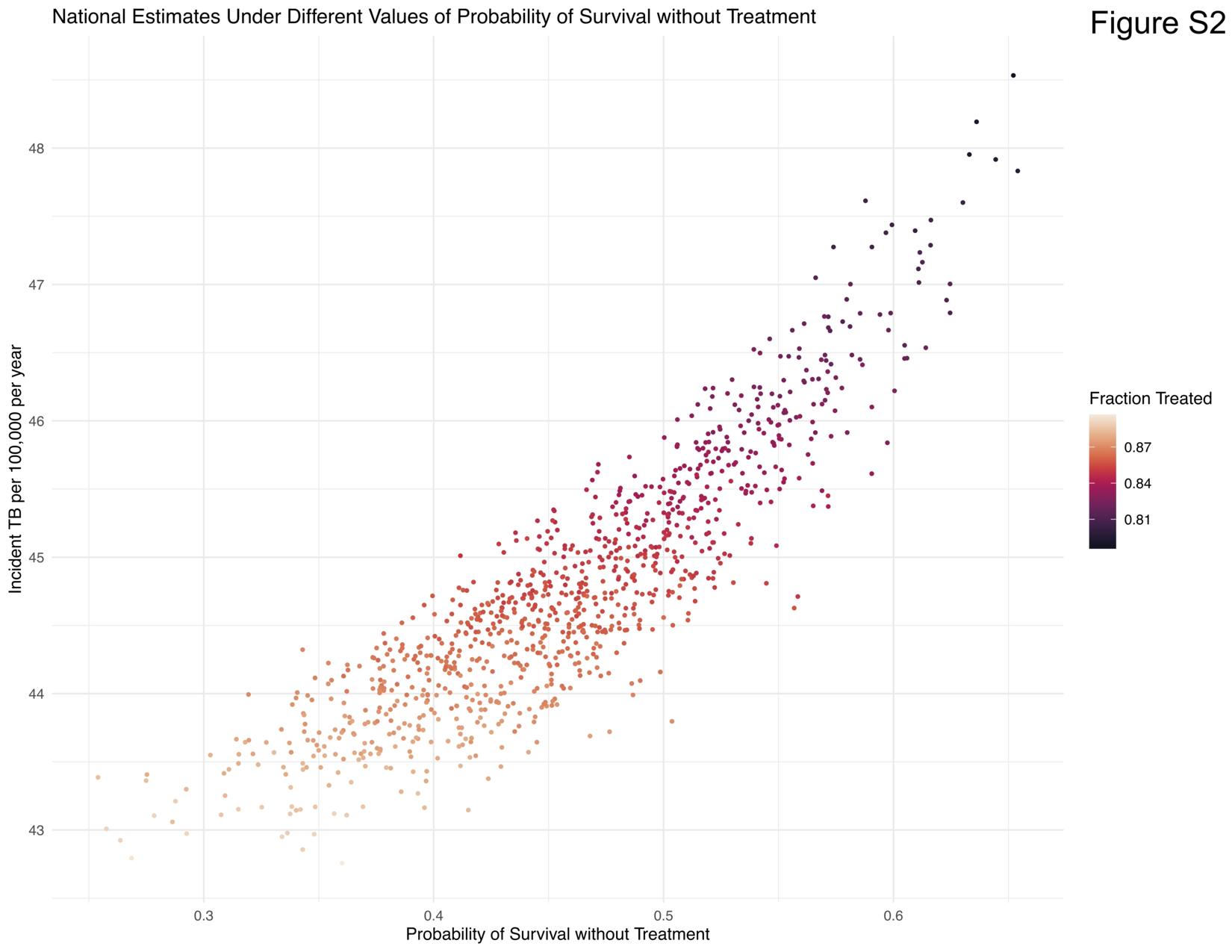

Supplement: S2 Fig — (TIFF) [file pgph.0000725.s002.tiff]

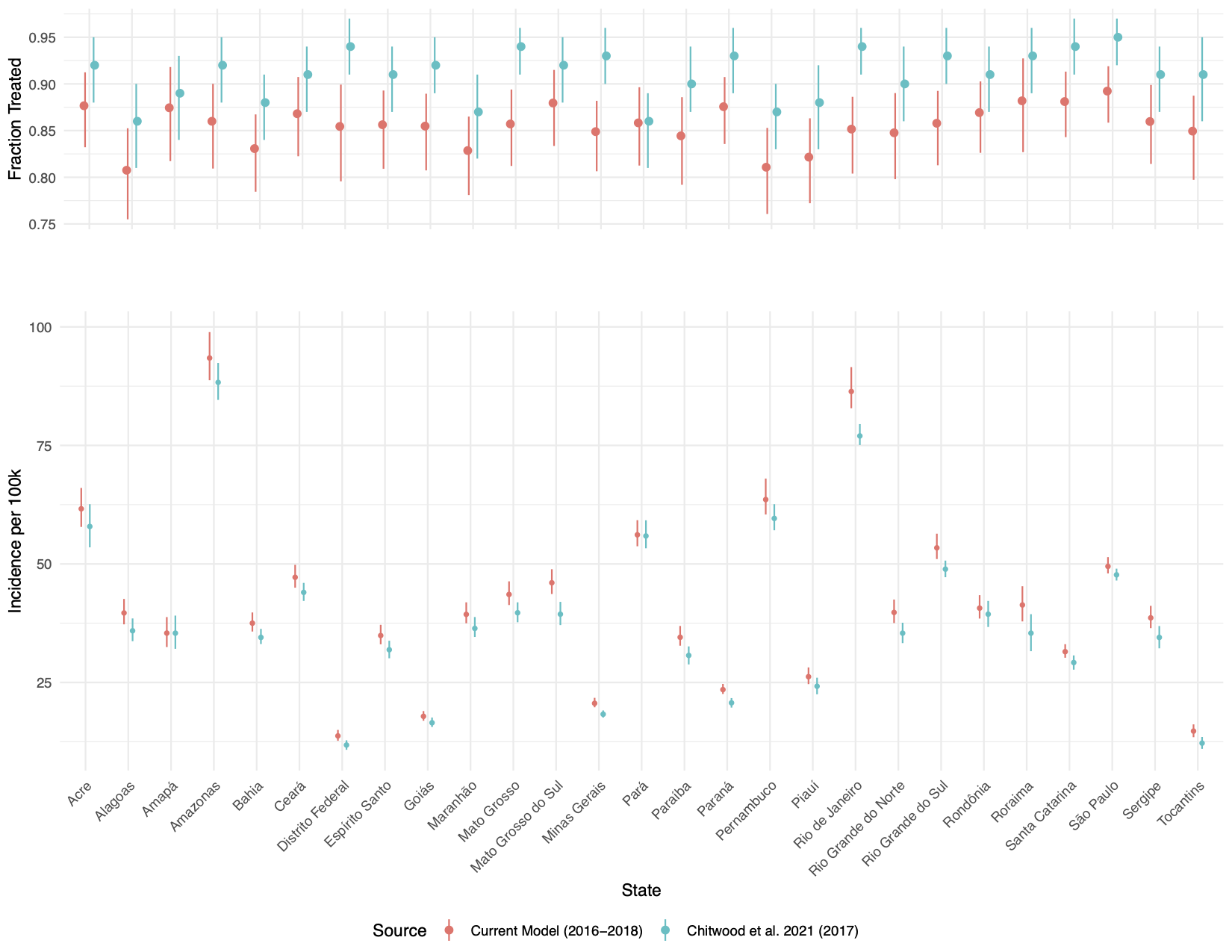

Supplement: S3 Fig — Comparison of state-level estimates of (A) incident TB per 100,000 population per year and (B) the fraction of individuals with incident TB receiving treatment estimated with the spatial-mechanistic model described in this manuscript (red) and previously published state-level estimates (blue). (TIFF) [file pgph.0000725.s003.tiff]
