# Supplementary material for: A spatial-mechanistic model to estimate subnational tuberculosis burden with routinely collected data: An application in Brazilian municipalities
Source: PLOS Glob Public Health. 2022 Sep 21;2(9):e0000725. doi: 10.1371/journal.pgph.0000725 (PMC10021638; doi:10.1371/journal.pgph.0000725)
Supplement: S1 Table — (DOCX) [file pgph.0000725.s004.docx]

**Table S1: Model Parameters**

| Parameter |  | Prior Distribution | | Source |
| --- | --- | --- | --- | --- |
| Incidence constant | π_0_ | Normal (0,10) | | Assumed |
| Incidence regression coefficients | π | Normal (0,10) | | Assumed |
| Incidence random effects | θ_π_ | Normal (0,1) | | Assumed |
| Incidence ρ | ρ_π_ | Beta (1.5, 1.5) | | Assumed |
| Incidence spatial and random effects standard deviation | σ_π_ | Cauchy (0,2) | | Assumed |
| Fraction Treated constant | ω_0_ | Normal (0,10) | | Assumed |
| Fraction Treated regression coefficients | ω | Normal (0,10) | | Assumed |
| Fraction Treated random effects | θ_ω_ | Normal (0,1) | | Assumed |
| Fraction Treated ρ | ρ_ω_ | Beta (1.5, 1.5) | | Assumed |
| Fraction Treated spatial and random effects standard deviation | σ_ω_ | Cauchy (0,2) | | Assumed |
| Probability of surviving the disease episode without treatment | μ | Beta (25.7, 33.3) | Mean: 0.44  SD: 0.06 | (1) |
| Probability an individual with treatment outcome “death” appears in SIM | λ | Beta(28.4, 11.6) | Mean: 0.71  SD: o.07 | (2) |
| Probability that an individual with treatment outcome of “lost to follow up” appears in SIM | η | Beta (2.14, 40.7) | Mean: 0.05  SD: 0.03 | (2) |
| Death adjustment constant | κ_1_ | Normal (0,1) | | Assumed |
| Death adjustment random effects | κ_2_ | Normal (0,1) | | Assumed |
| Death adjustment regression coefficient: fraction of SIM deaths that have a poorly-defined cause of death | κ_3_ | Normal (0,1) | | (1) |
| Death adjustment random effect standard deviation | σ_κ_ | Cauchy (0,2) | | Assumed |
| Parameters with Municipality-Specific Prior Distributions | | | | |
| Incidence spatial effects  Mean | φ_π_ | Normal $\left( 0, \left( -\frac{1}{2}* {\sum_{i \sim j} \left( \phi_{\pi,i}- \phi_{\pi,j} \right)}^{2} \right) \right)$  Normal (0, 0.001) | | (3, 4) |
| Fraction Treated spatial effects  Mean | φ_ω_ | Normal $\left( 0, \left( -\frac{1}{2} {*\sum_{i \sim j} \left( \phi_{\omega,i}- \phi_{\omega,j} \right)}^{2} \right) \right)$  Normal (0, 0.001) | | (3, 4) |
| Probability that death is listed as the treatment outcome | ζ_i_ | $\beta\left( 10\left( \frac{Death on Treatment_{i}}{Cases_{i}-Missing Outcome_{i}} \right), 10\left( 1-\frac{Death on Treatment_{i}}{Cases_{i}-Missing Outcome_{i}} \right) \right)$ | | (5) |
| Probability that loss to follow-up is listed as the treatment outcome | ι_i_ | $\beta\left( 10\left( \frac{Loss to Follow Up_{i}}{Cases_{i}-Missing Outcome_{i}} \right), 10\left( 1-\frac{Loss to Follow Up_{i}}{Cases_{i}-Missing Outcome_{i}} \right) \right)$ | | (5) |

1. Chitwood MH, Pelissari DM, Drummond Marques da Silva G, Bartholomay P, Rocha MS, Sanchez M, et al. Bayesian evidence synthesis to estimate subnational TB incidence: An application in Brazil. Epidemics. 2021;35:100443.

2. Bartholomay P, Oliveira GP, Pinheiro RS, Vasconcelos AM. [Improved quality of tuberculosis data using record linkage.]. Cad Saude Publica. 2014;30(11):2459-70.

3. Morris M, Wheeler-Martin K, Simpson D, Mooney SJ, Gelman A, DiMaggio C. Bayesian hierarchical spatial models: Implementing the Besag York Mollie model in stan. Spat Spatiotemporal Epidemiol. 2019;31:100301.

4. Morris M. bym2.stan[Source Code].

5. Sistema de Informação de Agravos de Notificação – Sinan: normas e rotinas, 2nd ed., [Internet]. Ministério da Saúde, Secretaria de Vigilância em Saúde. Available from: <http://bvsms.saude.gov.br/bvs/publicacoes/07_0098_M.pdf>.
