# Supplementary material for: A spatial-mechanistic model to estimate subnational tuberculosis burden with routinely collected data: An application in Brazilian municipalities
Source: PLOS Glob Public Health. 2022 Sep 21;2(9):e0000725. doi: 10.1371/journal.pgph.0000725 (PMC10021638; doi:10.1371/journal.pgph.0000725)
Supplement: S2 Table — (DOCX) [file pgph.0000725.s005.docx]

**Table S2: Model Parameters**

| Parameter |  | Prior Distribution | | Posterior Distribution |
| --- | --- | --- | --- | --- |
| Incidence constant | π_0_ | Normal (0,10) | Mean: 0  SD: 10 | Mean: 3.98  95% CI: 3.94, 4.04 |
| Incidence regression coefficients | π | Normal (0,10) | Mean: 0  SD: 10 |  |
| *Household Crowding* | |  |  | Mean: 0.014  95% CI: -0.02, 0.05 |
| *Subnormal Agglomerations* | |  |  | Mean: 0.01  95% CI: -0.02, 0.04 |
| *Population in Poverty* | |  |  | Mean: -0.08  95% CI: -0.12, -0.04 |
| *SUS Beds per capita* | |  |  | Mean: 0.03  95% CI: 0.01, 0.05 |
| *FHS Team Coverage* | |  |  | Mean: -0.01  95% CI: -0.04, 0.01 |
| *Prison in Municipality* | |  |  | Mean: 0.42  95% CI: 0.38, 0.47 |
| *GPD per capita* | |  |  | Mean: 0.00  95% CI: -0.02, 0.02 |
| Incidence random effects | θ_π_ | Normal (0,1) | Mean: 0  SD: 1 | Mean: 0.00  Range: -2.08, 4.01 |
| Incidence ρ | ρ_π_ | Beta (1.5, 1.5) | Mean: 0.5  SD: 0.25 | Mean: 0.81  95% CI: 0.76, 0.86 |
| Fraction Treated constant | ω_0_ | Normal (0,10) | Mean: 0  SD: 10 | Mean: 1.63  95% CI: 1.32, 1.89 |
| Fraction Treated regression coefficients | ω | Normal (0,10) | Mean: 0  SD: 10 |  |
| *Population in Poverty* | |  |  | Mean: -0.03  95% CI:-0.08, 0.03 |
| *SUS Beds per capita* | |  |  | Mean: -0.03  95% CI: -0.06, 0.02 |
| *FHS Team Coverage* | |  |  | Mean: 0.01  95% CI: -0.04, 0.09 |
| *Prison in Municipality* | |  |  | Mean: 0.27  95% CI: 0.19, 0.37 |
| *GPD per capita* | |  |  | Mean: -0.02  95% CI: -0.05, 0.01 |
| Fraction Treated random effects | θ_ω_ | Normal (0,1) | Mean: 0  SD: 1 | Mean: 0.00  Range: -0.35, 0.60 |
| Fraction Treated ρ | ρ_ω_ | Beta (1.5, 1.5) | Mean: 0.5  SD: 0.25 | Mean: 0.92  95% CI: 0.77, 0.99 |
| Probability of surviving the disease episode without treatment | μ | Beta (25.7, 33.3) | Mean: 0.44  SD: 0.06 | Mean: 0.46  95% CI: 0.33, 0.60 |
| Probability an individual with treatment outcome “death” appears in SIM | λ | Beta(28.4, 11.6) | Mean: 0.71  SD: 0.07 | Mean: 0.31  95% CI: 0.23, 0.38 |
| Probability that an individual with treatment outcome of “lost to follow up” appears in SIM | η | Beta (2.14, 40.7) | Mean: 0.05  SD: 0.03 | Mean: 0.02  95% CI: 0.003, 0.07 |
| Death adjustment constant | κ_1_ | Normal (0,1) | Mean: 0  SD: 1 | Mean: -1.79  95% CI: -2.02, -1.56 |
| Death adjustment random effects | κ_2_ | Normal (0,1) | Mean: 0  SD: 1 | Mean: 0.00  Range: -0.49, 1.63 |
| Death adjustment regression coefficient: fraction of SIM deaths that have a poorly-defined cause of death | κ_3_ | Normal (0,1) | Mean: 0  SD: 1 | Mean: 2.57  95% CI:1.32, 3.80 |
