# Supplementary material for: A spatial-mechanistic model to estimate subnational tuberculosis burden with routinely collected data: An application in Brazilian municipalities
Source: PLOS Glob Public Health. 2022 Sep 21;2(9):e0000725. doi: 10.1371/journal.pgph.0000725 (PMC10021638; doi:10.1371/journal.pgph.0000725)
Supplement: S3 Table — (DOCX) [file pgph.0000725.s006.docx]

**Table S2:** Municipalities with the Highest TB Incidence Rates

| Municipality | State | Cases | Pop. | Prison in Mun. | Incidence (100,000/Year) | Fraction Treated | Untreated TB (100,000/Year) |
| --- | --- | --- | --- | --- | --- | --- | --- |
| BALBINOS | SÃO PAULO | 182 | 5242 | TRUE | 1172.83  (1009.31, 1360.6) | 0.94 (0.88, 0.97) | 3.8 (1.53, 7.71) |
| LAVÍNIA | SÃO PAULO | 329 | 11233 | TRUE | 988.44  (884.74, 1107.38) | 0.95 (0.9, 0.98) | 5.9 (2.53, 11.48) |
| PACAEMBU | SÃO PAULO | 273 | 14080 | TRUE | 665.13  (581.59, 754.05) | 0.95 (0.9, 0.98) | 5.02 (2.09, 9.68) |
| ILHA DE ITAMARACÁ | PERNAMBUCO | 395 | 25657 | TRUE | 555.44  (487.87, 639.76) | 0.9 (0.81, 0.96) | 14.05 (5, 30.21) |
| MARABÁ PAULISTA | SÃO PAULO | 98 | 5631 | TRUE | 541.21  (438.13, 650.6) | 0.93 (0.88, 0.96) | 2.28 (1.13, 3.92) |
| ÁLVARO DE CARVALHO | SÃO PAULO | 88 | 5124 | TRUE | 536.17  (418.56, 654.97) | 0.92 (0.87, 0.96) | 2.2 (1.12, 3.74) |
| PRACINHA | SÃO PAULO | 56 | 3799 | TRUE | 437.15  (325.34, 567.11) | 0.94 (0.89, 0.97) | 1 (0.41, 1.82) |
| PRESIDENTE BERNARDES | SÃO PAULO | 178 | 13368 | TRUE | 435.01  (371.16, 505.71) | 0.93 (0.89, 0.97) | 3.83 (1.88, 6.51) |
| ITAPISSUMA | PERNAMBUCO | 308 | 26269 | TRUE | 428.61  (368.6, 497.44) | 0.89 (0.81, 0.95) | 12.19 (4.93, 24.29) |
| IRAPURU | SÃO PAULO | 101 | 8242 | TRUE | 403.38  (330.62, 487.16) | 0.95 (0.9, 0.98) | 1.8 (0.77, 3.41) |
| TUPI PAULISTA | SÃO PAULO | 180 | 15321 | TRUE | 390.82  (335.61, 452.18) | 0.94 (0.9, 0.97) | 3.67 (1.6, 6.65) |
| FLORÍNIA | SÃO PAULO | 43 | 2745 | TRUE | 387.18  (271.56, 527.96) | 0.92 (0.88, 0.96) | 0.82 (0.39, 1.48) |
| CAMPINÁPOLIS | MATO GROSSO | 156 | 15489 | FALSE | 352.26  (293.04, 418.01) | 0.85 (0.78, 0.91) | 8.06 (4.32, 13.12) |
| POTIM | SÃO PAULO | 234 | 23471 | TRUE | 343.84  (298.1, 400.13) | 0.92 (0.84, 0.97) | 6.94 (2.65, 14.18) |
| ITAITINGA | CEARÁ | 362 | 39254 | TRUE | 329.34  (288.85, 375.16) | 0.9 (0.83, 0.94) | 13.15 (6.75, 22.99) |
| GETULINA | SÃO PAULO | 112 | 11352 | TRUE | 316.33  (262.73, 377.02) | 0.92 (0.87, 0.95) | 2.94 (1.54, 4.6) |
| MIRANDÓPOLIS | SÃO PAULO | 258 | 29305 | TRUE | 302.54  (264.59, 347.18) | 0.94 (0.9, 0.97) | 5.1 (2.32, 9.42) |
| CHARQUEADAS | RIO GRANDE DO SUL | 327 | 39266 | TRUE | 298.69  (262.29, 346.39) | 0.9 (0.83, 0.95) | 11.58 (5.3, 21.82) |
| VALPARAÍSO | SÃO PAULO | 212 | 25693 | TRUE | 278.3  (240.43, 319.54) | 0.94 (0.9, 0.97) | 4 (1.74, 7.29) |
| PIRAJUÍ | SÃO PAULO | 194 | 24998 | TRUE | 270.64  (231.73, 309.44) | 0.91 (0.86, 0.95) | 6.28 (3.45, 10.45) |
| JUNQUEIRÓPOLIS | SÃO PAULO | 162 | 20363 | TRUE | 266.51  (222.31, 314.27) | 0.94 (0.9, 0.97) | 3.29 (1.56, 5.79) |
| SERRA AZUL | SÃO PAULO | 109 | 13887 | TRUE | 260.44  (211.84, 313.18) | 0.9 (0.84, 0.94) | 3.76 (1.86, 6.32) |
| JACAREACANGA | PARÁ | 64 | 8339 | FALSE | 258.53  (200.88, 328.45) | 0.84 (0.76, 0.9) | 3.51 (1.99, 5.82) |
| GUAREÍ | SÃO PAULO | 136 | 17636 | TRUE | 252.97  (208.56, 301.41) | 0.92 (0.87, 0.96) | 3.42 (1.59, 6.36) |
| TREMEMBÉ | SÃO PAULO | 323 | 45987 | TRUE | 246.19  (217.95, 279.51) | 0.92 (0.86, 0.97) | 8.87 (3.75, 17.69) |
| FLÓRIDA PAULISTA | SÃO PAULO | 104 | 14304 | TRUE | 244.87  (200.61, 293.67) | 0.94 (0.9, 0.97) | 2.06 (0.89, 3.81) |
| MARTINÓPOLIS | SÃO PAULO | 188 | 26126 | TRUE | 240.14  (207.07, 277.94) | 0.94 (0.9, 0.97) | 3.95 (1.98, 6.47) |
| REGINÓPOLIS | SÃO PAULO | 70 | 9095 | TRUE | 237.21  (182.85, 299.79) | 0.91 (0.87, 0.95) | 1.87 (0.92, 3.14) |
| SANTA IZABEL DO PARÁ | PARÁ | 443 | 68756 | TRUE | 232.8  (207.07, 262.66) | 0.9 (0.83, 0.95) | 16.07 (7.54, 29.03) |
| MONGAGUÁ | SÃO PAULO | 338 | 54457 | TRUE | 220.45  (194.69, 246.13) | 0.93 (0.88, 0.96) | 9 (4.7, 15.28) |
| NÍSIA FLORESTA | RIO GRANDE DO NORTE | 169 | 27209 | TRUE | 219.08  (182.04, 261.52) | 0.89 (0.81, 0.95) | 6.66 (2.76, 12.98) |
| RIOLÂNDIA | SÃO PAULO | 82 | 12119 | TRUE | 215.8  (170.81, 264.06) | 0.91 (0.85, 0.95) | 2.44 (1.17, 4.21) |
| BERNARDINO DE CAMPOS | SÃO PAULO | 69 | 11162 | TRUE | 201.73  (157.7, 250.48) | 0.92 (0.86, 0.96) | 1.81 (0.81, 3.25) |
| CERQUEIRA CÉSAR | SÃO PAULO | 112 | 19524 | TRUE | 189.91  (157.35, 226.7) | 0.92 (0.87, 0.96) | 2.92 (1.46, 4.92) |
| LUCÉLIA | SÃO PAULO | 117 | 21465 | TRUE | 183.7  (150.59, 218.69) | 0.94 (0.9, 0.97) | 2.33 (1.11, 4.08) |
| SÃO VICENTE | SÃO PAULO | 1656 | 360514 | TRUE | 168.5  (158.8, 179.36) | 0.91 (0.87, 0.94) | 55.84 (34.2, 85.86) |
| CAPELA DO ALTO | SÃO PAULO | 102 | 20057 | TRUE | 166.79  (136.75, 200.63) | 0.92 (0.88, 0.96) | 2.58 (1.23, 4.55) |
| SÃO PEDRO DE ALCÂNTARA | SANTA CATARINA | 30 | 5600 | TRUE | 165.84  (110.62, 233.62) | 0.87 (0.79, 0.94) | 1.21 (0.5, 2.33) |
| TAQUARITUBA | SÃO PAULO | 102 | 23207 | TRUE | 150.43  (122.5, 178.97) | 0.92 (0.86, 0.96) | 2.88 (1.44, 5.01) |
