# Supplementary material for: A spatial-mechanistic model to estimate subnational tuberculosis burden with routinely collected data: An application in Brazilian municipalities
Source: PLOS Glob Public Health. 2022 Sep 21;2(9):e0000725. doi: 10.1371/journal.pgph.0000725 (PMC10021638; doi:10.1371/journal.pgph.0000725)
Supplement: S4 Table — (DOCX) [file pgph.0000725.s007.docx]

**Table S3:** Municipalities with the Highest Number of Individuals with Untreated TB

| Municipality | State | Incidence (100,000/Year) | Fraction Treated | Untreated TB (100,000/Year) | Untreated TB (Cases/Year) |
| --- | --- | --- | --- | --- | --- |
| RIO BRANCO | ACRE | 85.45 (79.46, 92.54) | 0.89 (0.84, 0.93) | 9.46 (5.86, 14.37) | 141.74 (87.92, 215.48) |
| MACEIÓ | ALAGOAS | 61.2 (56.26, 66.76) | 0.82 (0.76, 0.88) | 10.96 (6.82, 15.74) | 1142.81 (710.74, 1640.59) |
| MANAUS | AMAZONAS | 129.06 (121.88, 137.45) | 0.87 (0.81, 0.91) | 17.23 (10.57, 25.61) | 7768.63 (4763.59, 11548.47) |
| FEIRA DE SANTANA | BAHIA | 39.5 (35.95, 43.55) | 0.86 (0.81, 0.91) | 5.4 (3.37, 7.98) | 207.74 (129.48, 306.77) |
| ITABUNA | BAHIA | 75.88 (67.95, 84.71) | 0.84 (0.77, 0.89) | 12.28 (7.72, 18.84) | 58.33 (36.66, 89.51) |
| SALVADOR | BAHIA | 66.92 (62.52, 71.94) | 0.85 (0.79, 0.9) | 10.32 (6.27, 14.93) | 8776.79 (5332.96, 12699.32) |
| CAUCAIA | CEARÁ | 60.07 (54.09, 66.3) | 0.87 (0.81, 0.92) | 7.75 (4.34, 12.08) | 101.25 (56.78, 157.84) |
| FORTALEZA | CEARÁ | 74.13 (69.08, 79.97) | 0.87 (0.81, 0.93) | 9.56 (4.88, 15.15) | 6595.91 (3365.97, 10453.64) |
| BRASÍLIA | DISTRITO FEDERAL | 13.73 (12.7, 14.99) | 0.85 (0.8, 0.9) | 2.01 (1.32, 2.97) | 1804.53 (1185.42, 2667.47) |
| SERRA | ESPÍRITO SANTO | 42.23 (38.31, 47) | 0.86 (0.79, 0.92) | 5.88 (3.24, 9.46) | 147.92 (81.43, 237.75) |
| VILA VELHA | ESPÍRITO SANTO | 42.7 (38.68, 46.99) | 0.87 (0.81, 0.93) | 5.41 (2.91, 8.59) | 126.86 (68.22, 201.31) |
| VITÓRIA | ESPÍRITO SANTO | 43.82 (38.89, 49.3) | 0.84 (0.76, 0.9) | 7.28 (4.18, 11.41) | 94.44 (54.25, 148.06) |
| APARECIDA DE GOIÂNIA | GOIÁS | 38.96 (35.38, 43.16) | 0.87 (0.82, 0.92) | 4.95 (3, 7.54) | 148.02 (89.69, 225.43) |
| GOIÂNIA | GOIÁS | 20.49 (18.71, 22.68) | 0.85 (0.79, 0.91) | 3.01 (1.82, 4.71) | 650.85 (393.98, 1019.02) |
| SÃO LUÍS | MARANHÃO | 82.57 (76.72, 89.52) | 0.85 (0.79, 0.9) | 12.82 (7.73, 18.92) | 1522.4 (918.67, 2246.77) |
| CUIABÁ | MATO GROSSO | 77.38 (71.11, 84.65) | 0.86 (0.8, 0.91) | 11.16 (6.36, 17.16) | 394.21 (224.56, 605.95) |
| CAMPO GRANDE | MATO GROSSO DO SUL | 51.05 (47.01, 55.75) | 0.88 (0.83, 0.93) | 5.95 (3.27, 9.38) | 455.3 (250.23, 717.63) |
| BELO HORIZONTE | MINAS GERAIS | 26.6 (24.79, 28.75) | 0.86 (0.81, 0.91) | 3.7 (2.31, 5.48) | 2336.91 (1455.85, 3458.88) |
| JUIZ DE FORA | MINAS GERAIS | 53.48 (48.65, 58.7) | 0.86 (0.8, 0.91) | 7.71 (4.61, 11.54) | 243.92 (145.9, 365.4) |
| ANANINDEUA | PARÁ | 83.84 (76.68, 91.96) | 0.86 (0.8, 0.92) | 11.61 (6.18, 17.96) | 311.01 (165.38, 480.87) |
| BELÉM | PARÁ | 115.04 (107.02, 125.26) | 0.86 (0.8, 0.92) | 15.84 (8.63, 25.26) | 3382.37 (1842.91, 5394.03) |
| JOÃO PESSOA | PARAÍBA | 56.87 (52.35, 62.23) | 0.86 (0.79, 0.91) | 8.27 (4.87, 13.21) | 535.33 (314.99, 854.93) |
| CURITIBA | PARANÁ | 21.24 (19.53, 23.35) | 0.88 (0.81, 0.92) | 2.63 (1.53, 4.45) | 956.17 (555.05, 1616.58) |
| CABO DE SANTO AGOSTINHO | PERNAMBUCO | 63.1 (54.94, 72.25) | 0.77 (0.68, 0.85) | 14.79 (8.88, 22.18) | 61.67 (37.02, 92.5) |
| CARUARU | PERNAMBUCO | 62.15 (56.1, 69.17) | 0.83 (0.77, 0.88) | 10.59 (6.7, 15.32) | 133.38 (84.43, 193.03) |
| JABOATÃO DOS GUARARAPES | PERNAMBUCO | 76.66 (69.16, 85.02) | 0.79 (0.72, 0.86) | 16.48 (9.98, 24.02) | 796.01 (481.87, 1160) |
| OLINDA | PERNAMBUCO | 94.1 (84.09, 106.41) | 0.78 (0.7, 0.86) | 20.8 (11.71, 31.58) | 317.79 (178.89, 482.54) |
| PAULISTA | PERNAMBUCO | 69.48 (61.88, 77.86) | 0.79 (0.71, 0.86) | 14.76 (9.26, 21.97) | 158.56 (99.49, 235.94) |
| RECIFE | PERNAMBUCO | 116.48 (107.54, 127.66) | 0.82 (0.75, 0.88) | 21.09 (12.64, 31.86) | 5620.71 (3366.79, 8489.89) |
| TERESINA | PIAUÍ | 36.14 (32.9, 39.51) | 0.84 (0.78, 0.89) | 5.87 (3.6, 8.47) | 426.84 (261.79, 616.12) |
| BELFORD ROXO | RIO DE JANEIRO | 79.52 (71.15, 89.2) | 0.8 (0.71, 0.87) | 16.38 (9.75, 25.08) | 408.73 (243.25, 625.81) |
| CAMPOS DOS GOYTACAZES | RIO DE JANEIRO | 69.59 (63.64, 75.68) | 0.87 (0.82, 0.91) | 9.38 (5.63, 13.59) | 228.61 (137.3, 331.26) |
| DUQUE DE CAXIAS | RIO DE JANEIRO | 103.78 (95.84, 112.7) | 0.81 (0.75, 0.87) | 19.42 (12.62, 27.9) | 1564.13 (1016.17, 2247.43) |
| MAGÉ | RIO DE JANEIRO | 85.02 (76.21, 94.94) | 0.85 (0.78, 0.91) | 12.83 (7.13, 20.29) | 73.39 (40.81, 116.04) |
| MESQUITA | RIO DE JANEIRO | 90.96 (81.29, 102.31) | 0.83 (0.76, 0.88) | 15.51 (9.93, 23.13) | 46.22 (29.59, 68.93) |
| NITERÓI | RIO DE JANEIRO | 61.74 (55.17, 68.57) | 0.85 (0.77, 0.92) | 9.41 (4.43, 15.12) | 237.97 (112.04, 382.29) |
| NOVA IGUAÇU | RIO DE JANEIRO | 96.91 (89.7, 105.04) | 0.83 (0.78, 0.88) | 16.36 (10.91, 23.56) | 1060.18 (706.74, 1526.69) |
| RIO DE JANEIRO | RIO DE JANEIRO | 115.61 (110, 122.39) | 0.86 (0.82, 0.91) | 15.69 (10.1, 22.36) | 67715.31 (43605.12, 96501.44) |
| SÃO GONÇALO | RIO DE JANEIRO | 63.04 (57.74, 69.12) | 0.85 (0.78, 0.92) | 9.27 (4.62, 14.85) | 1035.6 (516.51, 1659.48) |
| SÃO JOÃO DE MERITI | RIO DE JANEIRO | 103.09 (94.58, 113.2) | 0.82 (0.75, 0.88) | 18.84 (11.83, 27.62) | 406.12 (255.13, 595.42) |
| NATAL | RIO GRANDE DO NORTE | 59.73 (54.53, 66.03) | 0.85 (0.78, 0.92) | 8.74 (4.63, 14.12) | 677.09 (358.98, 1094) |
| ALVORADA | RIO GRANDE DO SUL | 122.7 (111.48, 135.78) | 0.85 (0.78, 0.9) | 18.89 (11.15, 28.95) | 82 (48.4, 125.65) |
| CANOAS | RIO GRANDE DO SUL | 73.07 (66.54, 80.19) | 0.87 (0.81, 0.93) | 9.33 (5.02, 15.02) | 110.28 (59.4, 177.5) |
| CAXIAS DO SUL | RIO GRANDE DO SUL | 45.8 (41.67, 50.53) | 0.88 (0.82, 0.92) | 5.62 (3.33, 8.59) | 134.21 (79.58, 205.22) |
| PELOTAS | RIO GRANDE DO SUL | 63.6 (57.29, 69.95) | 0.87 (0.8, 0.92) | 8.6 (4.65, 13.47) | 101.31 (54.76, 158.73) |
| PORTO ALEGRE | RIO GRANDE DO SUL | 108.05 (100.67, 118.41) | 0.86 (0.79, 0.91) | 14.83 (8.57, 24.47) | 3256.7 (1881.59, 5372.01) |
| RIO GRANDE | RIO GRANDE DO SUL | 105.92 (94.44, 120.02) | 0.84 (0.74, 0.91) | 17.49 (8.27, 29.99) | 76.63 (36.23, 131.34) |
| VIAMÃO | RIO GRANDE DO SUL | 74.69 (66.53, 83.99) | 0.83 (0.75, 0.9) | 12.5 (7.28, 20.14) | 80.4 (46.81, 129.51) |
| PORTO VELHO | RONDÔNIA | 84.69 (79.16, 91.01) | 0.88 (0.84, 0.92) | 10.09 (6.8, 14.75) | 269.47 (181.46, 393.71) |
| FLORIANÓPOLIS | SANTA CATARINA | 53.7 (49.07, 58.79) | 0.89 (0.83, 0.94) | 6.16 (3, 9.89) | 145.24 (70.62, 233.17) |
| JOINVILLE | SANTA CATARINA | 41.29 (37.66, 45.44) | 0.89 (0.83, 0.93) | 4.61 (2.57, 7.4) | 153.37 (85.32, 246.16) |
| CAMPINAS | SÃO PAULO | 39.33 (36.85, 42.33) | 0.9 (0.85, 0.93) | 4.02 (2.44, 6.14) | 562.55 (341.9, 859.3) |
| CARAPICUÍBA | SÃO PAULO | 62.95 (57.46, 68.56) | 0.89 (0.84, 0.93) | 7.13 (4.17, 10.71) | 112.12 (65.66, 168.46) |
| GUARUJÁ | SÃO PAULO | 116.73 (105.64, 129.68) | 0.86 (0.79, 0.92) | 16.82 (8.3, 26.99) | 167.71 (82.74, 269.05) |
| GUARULHOS | SÃO PAULO | 47.6 (44.4, 51.14) | 0.89 (0.84, 0.93) | 5.44 (3.2, 8.14) | 993.09 (583.32, 1484.47) |
| OSASCO | SÃO PAULO | 57.03 (53.08, 61.3) | 0.9 (0.85, 0.94) | 5.91 (3.34, 9.03) | 287.22 (162.45, 438.76) |
| PRAIA GRANDE | SÃO PAULO | 113.03 (104.01, 123.26) | 0.91 (0.85, 0.96) | 10.81 (4.66, 18.72) | 104.74 (45.12, 181.4) |
| RIBEIRÃO PRETO | SÃO PAULO | 38.62 (35.27, 42.51) | 0.86 (0.8, 0.92) | 5.32 (3.12, 8.26) | 248.92 (146.07, 385.96) |
| SANTO ANDRÉ | SÃO PAULO | 36.09 (32.96, 39.29) | 0.89 (0.84, 0.93) | 4.11 (2.49, 6.2) | 209.87 (126.98, 316.72) |
| SANTOS | SÃO PAULO | 99.6 (92.09, 107.98) | 0.87 (0.81, 0.91) | 13.49 (8.34, 20.06) | 254.09 (157.2, 378) |
| SÃO BERNARDO DO CAMPO | SÃO PAULO | 35.23 (32.63, 38.02) | 0.9 (0.86, 0.94) | 3.48 (2.11, 5.26) | 238.2 (144.8, 360.1) |
| SÃO JOSÉ DOS CAMPOS | SÃO PAULO | 31.55 (28.82, 34.51) | 0.88 (0.83, 0.92) | 3.71 (2.31, 5.72) | 184.11 (114.69, 283.79) |
| SÃO PAULO | SÃO PAULO | 62.05 (59.52, 65.42) | 0.9 (0.85, 0.94) | 6.33 (3.87, 9.56) | 92822.51 (56793.78, 140143.77) |
| SÃO VICENTE | SÃO PAULO | 168.5 (158.8, 179.36) | 0.91 (0.87, 0.94) | 15.49 (9.49, 23.82) | 201.37 (123.33, 309.6) |
| ARACAJU | SERGIPE | 44.39 (40.64, 48.96) | 0.87 (0.81, 0.92) | 5.77 (3.17, 9.11) | 241.58 (132.58, 381.26) |
